# Carbon Footprint and Energy Transformation Analysis of Steel Produced via a Direct Reduction Plant with an Integrated Electric Melting Unit

**DOI:** 10.1007/s40831-022-00585-x

**Published:** 2022-08-31

**Authors:** Julian Suer, Frank Ahrenhold, Marzia Traverso

**Affiliations:** 1 Institute of Sustainability in Civil Engineering, RWTH Aachen University, Mies-van-der-Rohe-Str. 1, Aachen, Germany; 2 Thyssenkrupp Steel Europe AG, Duisburg, Germany

**Keywords:** Carbon footprint, Direct reduction plants, Electric melting unit, Energy transformation, Hydrogen, Integrated steel site

## Abstract

The production of flat steel products is commonly linked to highly integrated sites, which include hot metal generation via the blast furnace, basic oxygen furnace (BOF), continuous casting, and subsequent hot-rolling. In order to reach carbon neutrality a shift away from traditional carbon-based metallurgy is required within the next decades. Direct reduction (DR) plants are capable to support this transition and allow even a stepwise reduction in CO_2_ emissions. Nevertheless, the implementation of these DR plants into integrated metallurgical plants includes various challenges. Besides metallurgy, product quality, and logistics, special attention is given on future energy demand. On the basis of carbon footprint methodology (ISO 14067:2019) different scenarios of a stepwise transition are evaluated and values of possible CO_2_equivalent (CO_2_eq) reduction are coupled with the demand of hydrogen, electricity, natural gas, and coal. While the traditional blast furnace—BOF route delivers a surplus of electricity in the range of 0.7 MJ/kg hot-rolled coil; this surplus turns into a deficit of about 17 MJ/kg hot-rolled coil for a hydrogen-based direct reduction with an integrated electric melting unit. On the other hand, while the product carbon footprint of the blast furnace-related production route is 2.1 kg CO_2_eq/kg hot-rolled coil; this footprint can be reduced to 0.76 kg CO_2_eq/kg hot-rolled coil for the hydrogen-related route, provided that the electricity input is from renewable energies. Thereby the direct impact of the processes of the integrated site can even be reduced to 0.15 kg CO_2_eq/kg hot-rolled coil. Yet, if the electricity input has a carbon footprint of the current German or European electricity grid mix, the respective carbon footprint of hot-rolled coil even increases up to 3.0 kg CO_2_eq/kg hot-rolled coil. This underlines the importance of the availability of renewable energies.

**Graphical Abstract:**

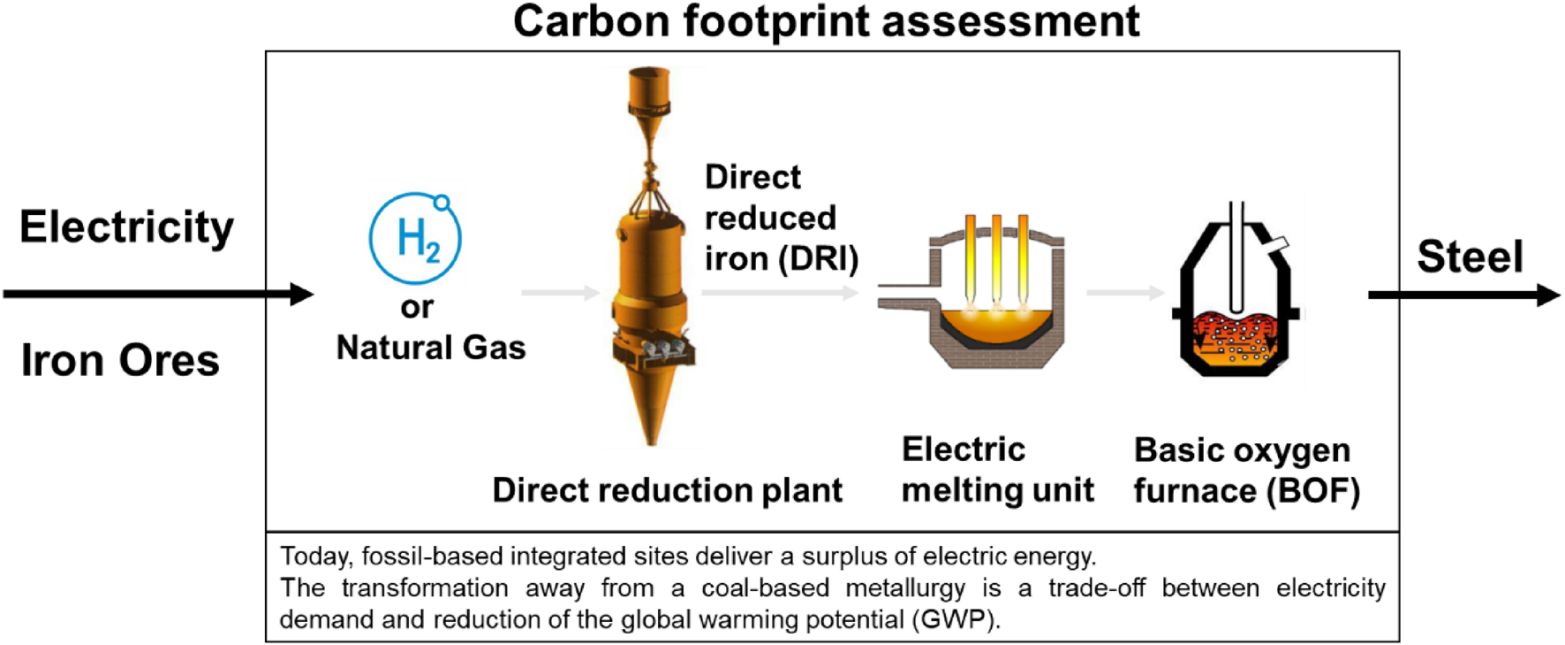

**Supplementary Information:**

The online version contains supplementary material available at 10.1007/s40831-022-00585-x.

## Introduction

The production of flat steel products is commonly linked to highly integrated sites. These sites normally include hot metal generation via the blast furnace, BOF, continuous casting, and subsequent hot-rolling. Including DR plants offers various new opportunities to these sites, especially a wide reduction in CO_2_eq emissions [1, 2]. Nevertheless, the implementation of DR plants into integrated metallurgical plants include various challenges. Metallurgical aspects need to be considered to maintain product quality, which reflects customer demand. Effects on the sites, internal and external energy network and on-site logistics must be evaluated and handled. Therefore, direct reduction with pure hydrogen and with natural gas as an interim solution combined with electrically melting are discussed.

Integrated steelmaking sites on the basis of blast furnace technology still account for 58% of steel production within the European Union (28) and even 73% of the worldwide steel is provided via the blast furnace route [3]. About 26% of the worldwide steel is produced by scrap recycling via an electric arc furnace (EAF) [3]. In sum, the energy-intensive steel industry is a large emitter of CO_2_ emissions accounting to about 7% of total worldwide anthropogenic emissions [4]. Although steel is a material with a highly effective recycling loop, the predicted worldwide demand of steel until 2050 and beyond needs considerable input of iron ore, since the increasing demand cannot be filled by scrap recycling alone [4].

It is presumed here that.

- Integrated sites persist to incorporate iron ore into the production cycle of steel.
- Integrated sites will continue to produce high purity steel qualities with superior surfaces, which set the standards in premium flat products.
- The coal-based metallurgy of blast furnaces within the integrated sites causes an inacceptable high carbon footprint. Coal-based reduction of ore needs to be replaced by carbon reduced techniques.

In previous years many different technologies have been suggested, which show the potential to make classical blast furnace technology obsolete. Most of these technologies need further development, and thus are incapable to start any transition process in time [5]. DR technology on the contrary is fully developed and commercially available. DR modules have now reached capacities, which allow replacing blast furnaces on a like for like basis. Modules above 2.5 Million tons of output per year are the state of the art already today, and future installations are likely to reach even higher capacities [1]. Although a pure hydrogen-based shaft furnace direct reduction process in a large scale has not been realized yet the concept is technically feasible and has already been proven for a large-scale hydrogen-rich (H_2_ content of 55–86%) shaft furnace direct reduction process [2].

**Fig. 1:**
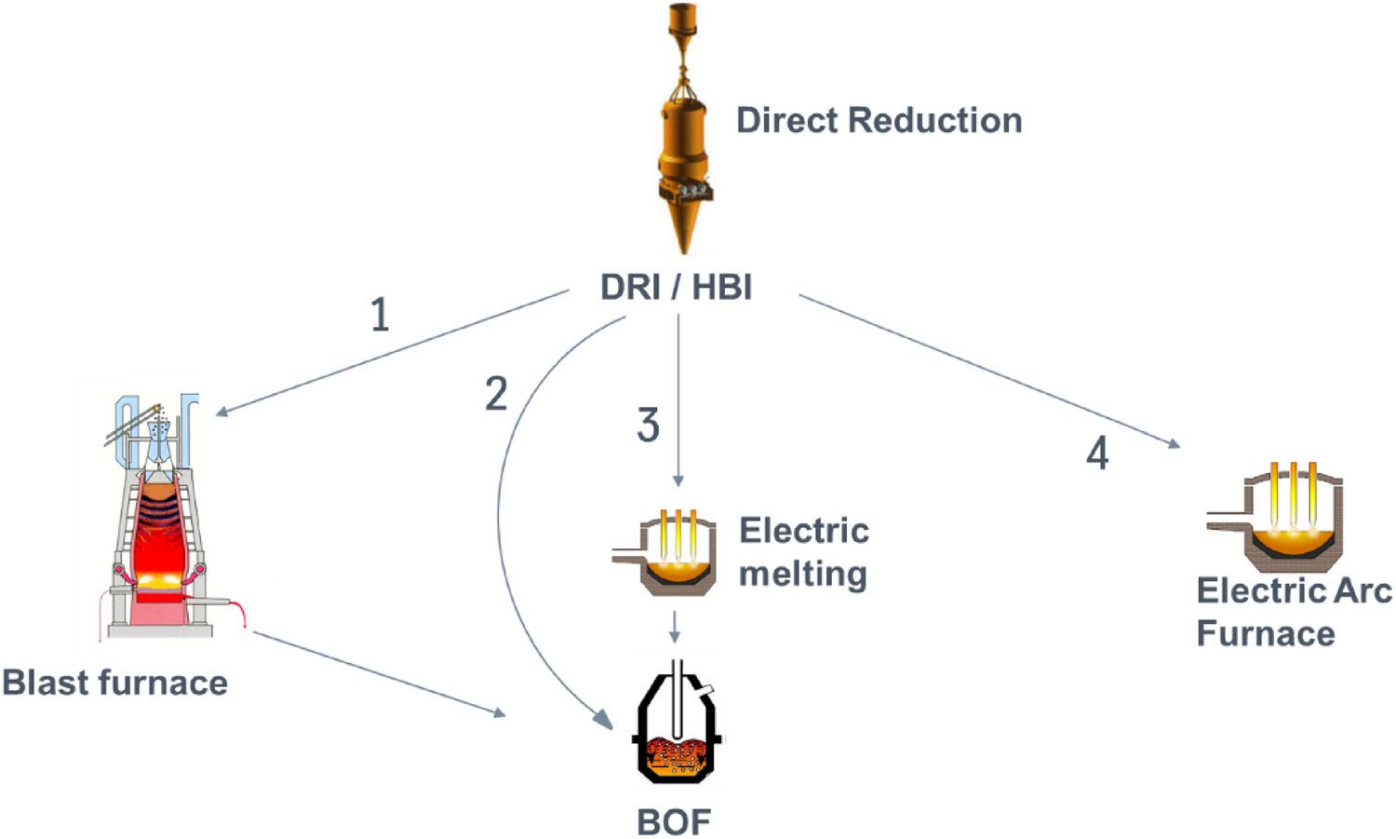
Possible Flow schemes for Direct reduced Iron/Hot briquetted Iron (DRI/HBI) at integrated sites

DR technology and direct reduced iron (DRI) material can be included into the existing material streams of existing plants in different ways. Figure 1 shows possible outbound material streams of DR plants**.** Several possible paths are described: the first one, DRI or in form of hot briquetted iron (HBI) material can provide feedstock to an existing blast furnace (BF), see arrow 1. HBI would be the natural choice in this case as DRI usage bears the risk of re-oxidation in the upper parts of the BF. Although the required carbon input into the blast furnace can be reduced by HBI input, the energy for melting still originates from coal. Subsequently reduction in carbon dioxide emissions is not complete [6].

Path 2 in Fig. 1 uses DRI or HBI as a scrap substitute at the BOF. Reduction in CO_2_eq emissions are limited as is the scrap rate in BOF steelmaking. Path 3 overcomes the limitations of path 2 by pre-melting DRI or HBI in an electric melting unit. This melt replaces hot metal and therefore makes blast furnaces obsolete. The melting unit process will still require some metallurgical carbon, which needs additional attention to reach decarbonized steel production. Path 4 uses a classical electric arc furnace (EAF) to melt DRI/HBI and scrap. This straightforward concept replaces not only blast furnaces but also BOFs. Some metallurgical carbon might be required here as well to preserve advantages of a foaming slag within the EAF [7].

In order to produce high quality steel grades lowest levels of nitrogen, phosphor, or carbon can be mandatory [8, 9]. Murphy discusses various aspects of nitrogen control in EAF steelmaking and concludes, “Technological solution is required to enable EAF to compete with BOF route on all grades” [8]. The problem to reach lowest nitrogen contents becomes even more difficult when lowest carbon content is simultaneously necessary [8, 9]. So far, no economically reasonable solution is available, while such steel grades are widely used in automotive applications, electro-mobility and deep drawing [9]. This can be a limitation for path 4 in Fig. 1 (EAF steelmaking).

In a direct comparison of converter vs. EAF steelmaking the following matters: The integrated steelmaking based on BOF process reaches nitrogen values between 20 and 40 ppm even in final products [9]. The BOF vessel shields the melt well against the surrounding atmosphere and it takes additional high-volume streams of carbon monoxide to keep nitrogen low throughout the blowing process. EAF modules do not present a similar air tightness and reach typical nitrogen values between 40 and 90 ppm [9].

Focus of this paper is the environmental evaluation of a DR plant combined with an electric melting unit (Fig. 1, path 3). The life cycle assessment (LCA) according to the international standards ISO 14040/44 [10, 11] is an established standardized methodology to determine the environmental influence of products. Within an LCA material and energy-related flows as well as environmental impacts are assessed in a holistic approach. LCAs for the current steel production are already widely applied in steel industry:

Norgate et al. [12], Burchart-Korol [13], Renzulli et al. [14], Chisalita et al. [15], and Backes et al. [16] presented LCAs for conventional steel production via the currently most common BF-BOF route. The presented product carbon footprints range from 1.6 kg CO_2_eq/kg steel up to 2.3 kg CO_2_eq/kg steel. Besides the product steel, some studies relate the environmental impact to the product hot-rolled coil. Different scrap rates, quality of raw materials, technical production sites, and methodological assumptions explain the differences.

LCAs for steel production via DR plants with electrically melting are not available in literature. Yet, there are environmental analyses with focus on carbon dioxide emissions and energy consumptions of steel production: Larsson et al. [17], Barati et al. [18], Harada and Tanka [19], Arens et al. [20], and Sarkar et al. [21] analyzed the carbon dioxide emissions and some of them the energy consumption of steel production via a natural gas-based direct reduction process combined with an EAF. Within the studies, the EAF is charged with different mixes of scrap and DRI. The carbon dioxide emissions range from 0.4 kg CO_2_/kg steel for an only scrap-based EAF operation up to 1.5 kg CO_2_/kg steel for an only DRI-based EAF operation. In the same way the reported energy consumptions range from 4 MJ/kg steel up to 23 MJ/kg steel. A steel production via a hydrogen-based direct reduction process combined with an EAF is presented by Fischedick et al. [22], Otto et al. [23], Vogl et al. [24], and Bhaskar et al. [25]. The carbon dioxide emissions depend significantly on the underlying grid emission factor of the used electricity mix.

Although most of the studies are comprehensive studies, none of these follow the LCA or product carbon footprint (PCF) methodology according to ISO 14040/44 [10, 11] and ISO 14067 [26], respectively. The presented study fills this gap by providing a holistic carbon footprint assessment according to ISO 14067 for this innovative steel production route and all environmental impacts from raw material acquisition to the product hot-rolled coil are included. In addition, the novel concept of incorporating an electric melting unit into integrated sites is discussed and analyzed, whereas the focus of the available literature is on classical EAFs.

**Fig. 2:**
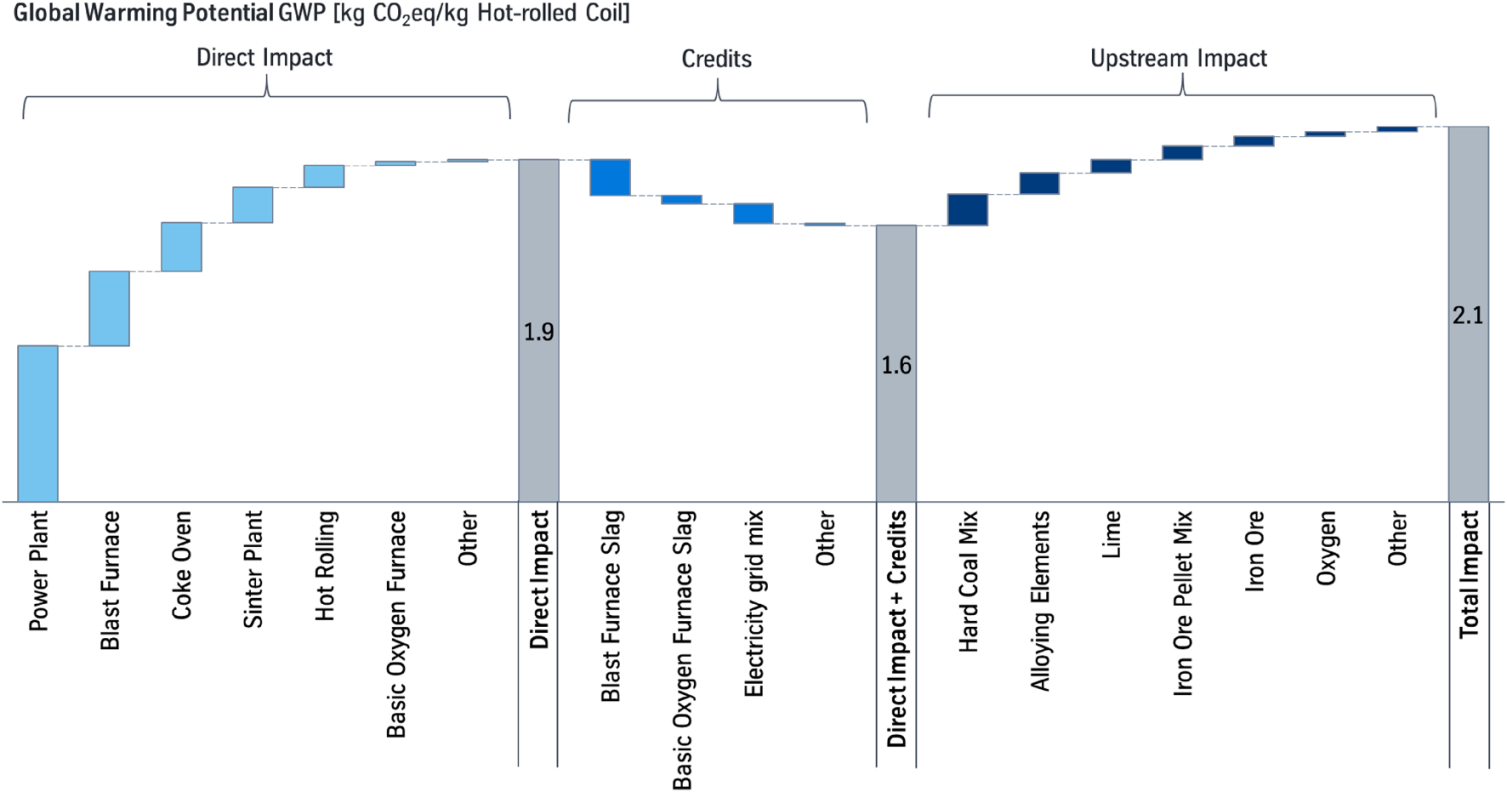
Global warming potential (GWP) of hot-rolled coil, produced over a conventional BF-BOF route (Base Case). Data base 2018 [27]

The presented study expands the study of Suer et al. [27], in which a PCF for hot-rolled coil produced via a conventional BF-BOF route is assessed. In Fig. 2, the results of the Base Case of the previous study are summarized.^1^ The Base Case of an integrated steel production via BF-BOF route amounts an overall carbon footprint of 2.1 kg CO_2_eq/kg hot-rolled coil. Individual contributions are split in sub-categories:

The direct impact describes the processes of the integrated steel site and add up to 1.9 kg CO_2_eq/kg hot-rolled coil (HRC). The impacts are attributed to the processes, where the respective emissions are emitted and not where they are caused. E.g., the impact of the power plant is caused by the processes, in which the process gases are produced, which are incinerated in the power plant. Turning off the power plant could not eliminate the emissions resulting from the process gases, but these would have to be incinerated somewhere else.

Following the principle of system expansion credits are given for the co-products [11, 26], which reduce the global warming potential (GWP) to about 1.6 kg CO_2_eq/kg HRC. Especially, the use of the blast furnace slag within the cement industry is an environmental useful cross-functional cooperation. These benefits need to be taken into account to avoid unnoticed shift of environmental impacts. The impact of the upstream processes add up to about 0.56 kg CO_2_eq/kg HRC [27]. The result of the previous study of 2.1 kg CO_2_eq/kg hot-rolled coil [27] is consistent to the carbon footprint from the GaBi database of 2.0 kg CO_2_eq/kg slab.^2^

In the previous study based on the results of the Base Case, modified BF operations are analyzed like the injection of hydrogen and the use of HBI in a BF. These measurements enable a reduced carbon input into the BF but the coke cannot be replaced, completely. Yet, the injection of hydrogen into existing blast furnaces can push the establishment of a hydrogen market and infrastructure and reduce the GHG emissions of the BF-BOF route. The use of HBI in a BF is a first step to integrate DR plants into an integrated steel site. [27]

Thus, these scenarios can function as intermediate scenarios towards a further CO_2_eq-reduced steel production. This goal is described in this paper by presenting a PCF for a natural gas-based and a hydrogen-based DR plant with an electric melting unit.

## Methodology

Since the data availability of future scenarios is not as technical mature as for conventional steel production, the focus of this paper lies on a single environmental impact category: climate change. Therefore the sum of greenhouse gas (GHG) emissions and removals of a product system, expressed as CO_2_eq are assessed. The mass of a GHG is converted into CO_2_eq by multiplying the mass of the GHG by the respective GWP. The GWP of a GHG characterizes its impact on the climate change in comparison to CO_2_. Since GHG have different life spans in the atmosphere a time horizon has to be defined. Within this paper the GWP 100 is used to represent the impact of the GHG emissions on climate change for a time horizon of 100 years. [26]

A carbon footprint of a product assessment according to ISO 14067 [26] is conform to an LCA according to ISO 14040 [10] and 14044 [11]. Whereas within an LCA several impact categories are assessed, the focus of a carbon footprint assessment is on the climate change as the single impact category [26]. The impact category climate change is a so-called midpoint category. The resulting effects from climate change, e.g. extreme weather events, are called endpoint categories [28] and are analysed e.g., by the IPCC [29].

Within this paper a so-called cradle-to-gate approach is followed. Thus, GHG emissions of a life cycle from mining of raw materials and energy carriers, transport, and production processes are included, which are required to produce the considered product [26]. The declared unit is 1 kg of hot-rolled coil. Further downstream treatment of the hot-rolled coil and the use phase are consciously excluded because steel products have several applications. Since the downstream treatment and the use phase are not affected by the considered scenarios, the cradle-to-gate approach is adequate to evaluate the impact of the scenarios on climate change.

The carbon footprint of all considered scenarios are based on the same methodology and databases.^3^ Following the methodology of a recycled content approach scrap does not have an environmental footprint and is considered as burden-free [30]. The emissions from scrap collection, sorting, and processing are not included in this study. For conventional steel production these emissions are ‘generally negligible’ [30]. This is also assumed for the future scenarios, which is a limitation of this study. However, this convention affects the absolute values of the scenarios but the relative differences between the scenarios are not affected, since the scrap input into the BOF is equal in all considered scenarios. The internal accumulated scrap until the product hot-rolled coil is recycled completely in the BOF.

For assessing the production of co-products, which are used outside the integrated steel mill, the method of system expansion is chosen. Thus, it is assumed that the co-product substitutes a primary production of the product and therefore a credit is given [11, 26]. Since the given credits depend on the environmental impacts of the substituted primarily produced products, these values have a degree of uncertainty when considering future scenarios. Therefore, like in Fig. 2 the individual contributions of the processes are presented in this paper so that each impact is transparent. Thus, the communicated PCF can also be converted into a PCF without the consideration of credits, which is also done in this paper.

Limitations of this study are that the data for the DR plant and the electric melting unit are based on metallurgical models from internal communication of thyssenkrupp Steel Europe AG (tkSE, 2020). However, technical primary data of a large scale shaft furnace direct reduction process in combination with an electric melting unit are not available. Incremental improvements for the future scenarios are not considered. Emissions from combustion processes of internal transportation and emissions from the construction phase of facilities, machines, and infrastructure of the integrated steel site are not included in this study. The cut-off criteria are conform to those defined by the Worldsteel Association [31]. Secondary data for inputs and co-products are taken from the GaBi software, database 2021.1 [32]. Further information and a list including all used GaBi databases are given in the supplementary materials of this paper.

## Product Carbon Footprint for a Natural Gas-Based Direct Reduction Plant with an Integrated Electric Melting Unit

### Goal and Scope

**Fig. 3:**
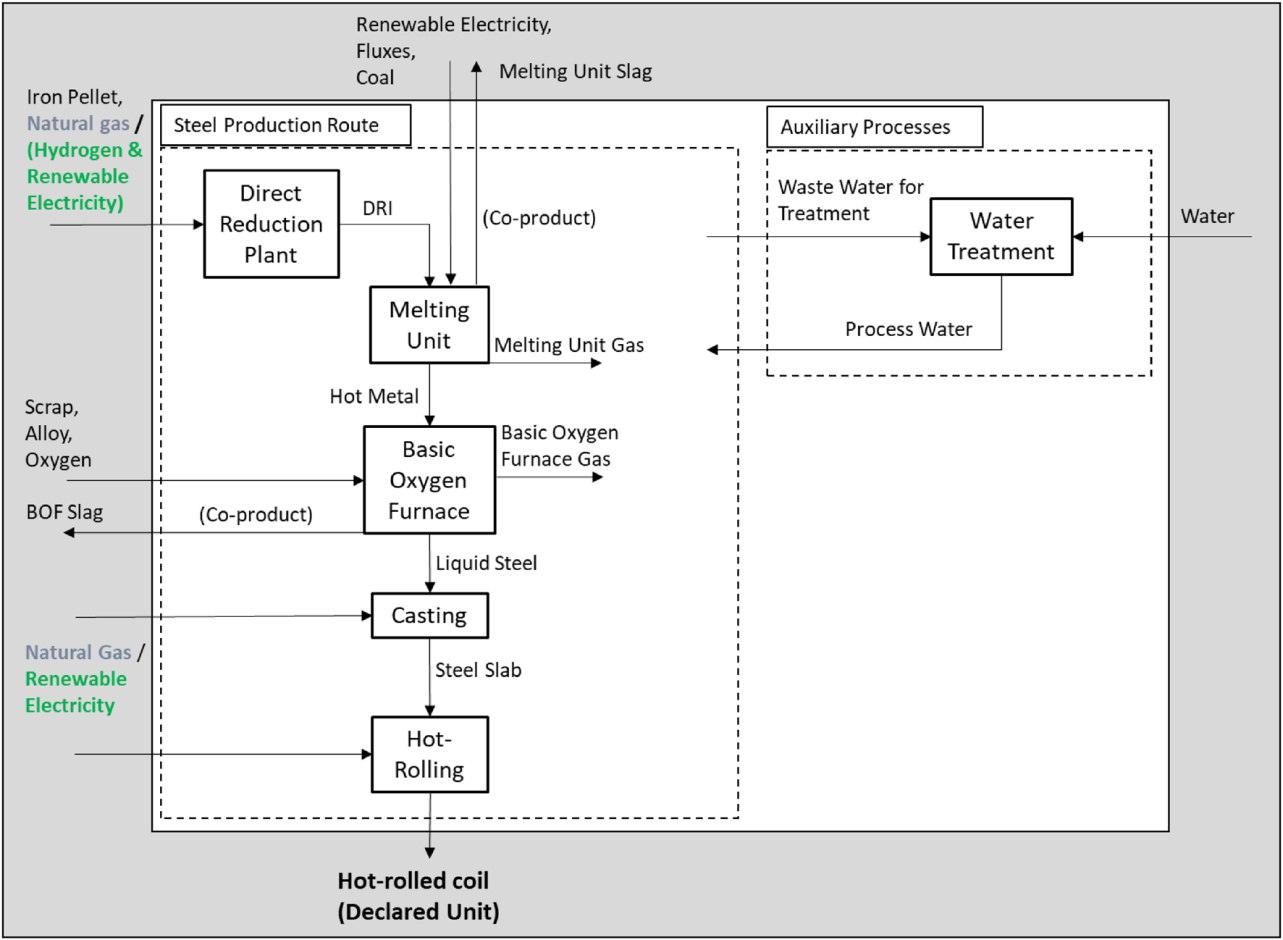
System boundary definition and major material streams of the future scenarios: natural gas (NG-Case; grey input) or hydrogen-based (H_2_-Case; green input) direct reduction with an integrated electric melting unit. White zone: processes of the integrated steel site. Grey zone: inputs and outputs of the integrated steel site. The process basic oxygen furnace (BOF) includes the secondary metallurgy. Not all considered inputs and outputs are listed in this figure for reasons of clarity

DR plants in combination with electric melting units are able to replace blast furnaces on a like for like basis. The outline of mass streams and boundaries is shown in Fig. 3 and matches Case 3 in Fig. 1, which uses a combination of electric melting and BOF technology. A carbon footprint assessment for the scenario natural gas-based (NG-Case) direct reduction with subsequently electrically melting is presented here. The inner boundary of Fig. 3 (white zone) includes the processes of the integrated site, the outer boundary (grey zone) includes the upstream materials and co-products, which are also considered within this study. The further downstream treatment of the hot-rolled coil or its use phases are excluded, since this carbon footprint assessment is a cradle-to-gate approach considering all processes until the product hot-rolled coil.

The product of the electric melting unit is an equal hot metal as the product from the BF. The hot metal is further refined within existing BOFs into crude steel. Thus, steel refining, secondary metallurgy, steel casting, and downstream processes do not need to change comparing a conventional integrated steel site.

The DR plant is fed exclusively with iron ore pellet feed. In general, the use of lump ore could be possible, as well. Subsequently, the DRI is charged hot into the electric melting unit. No co-product gas is generated from a DR plant. Although from the melting unit a carbon monoxide rich off-gas emerges, its amount is far below the range of the off-gases from the replaced BF. In sum, every DR plant in combination with an electric melting unit replacing a BF needs additional, newly generated electricity. Thus, the electricity surplus of the conventional BF-BOF route turns into a deficit for the DRI-based route.

Within the NG-Case, natural gas is used for the gas preheater of the DR plant and for the slab heating of the hot-rolling process, see Fig. 3. The electric melting unit and the BOF produce a carbon-monoxide rich off-gas. This could be converted into chemical products like methanol [33]. At least the process gases could be used for thermal heat supply. It is assumed that the process gases replace natural gas energetically one by one. Therefore, credit is given for the replacement of heat supply by natural gas. Since it is not sure, in which processes the off-gases will be used, the emissions, which result from incineration of the process gases, are attributed to the processes, in which the gases are produced: BOF and melting unit. The other emissions are attributed to those processes where they are emerged.

In order to keep up the useful cooperation between the steel and the cement industry, the produced slag from the electric melting unit should be able to substitute cement. It is a necessity that this slag adjustment is a goal of research activities. In this paper, it is assumed that the electric melting unit’s slag has the same characteristics like the blast furnace’s slag so that identical specific credit [kg CO_2_eq/kg slag] is given for the co-product. The GWP without this credit is also presented.

### Life Cycle Inventory

The data for the NG-Case are taken from internal communication of tkSE (2020). The cut-off criteria are conform to those defined by the Worldsteel Association [31]. Further explanation is given in the supplementary materials. The iron and energy feedstock of the integrated steel site are listed in Table 1. Other inputs like alloying elements, oxygen or fluxes (Fig. 3) are not listed in the table but considered in the carbon footprint assessment according to the defined cut-off criteria. The listed data are the most relevant for a comparison of the made scenarios.

**Table 1:** Major inputs of the integrated steel site for the NG-Case

Input	[Unit input/kg hot-rolled coil]
Iron ore pellets (kg)	1.5
Scrap (kg)	0.2
Natural gas (MJ)^a^	13
Electricity (MJ)	2.7
Coal (kg)	0.015

^a^Related to lower heating value (LHV) of 43.3 MJ/kg

The major energetic input is shifted from coal (Base Case, [27]) to natural gas. The scrap input is kept constant for reasons of comparability. Imported electricity is modelled as a German renewable electricity mix from the year 2018 according to the Environment Agency [34]. As renewable energy sources electricity from wind power, photovoltaics, biogas, biomass, hydropower, and geothermal energy are used. The construction of e.g., photovoltaics or windmills requires fossil energy. For the renewable energies the GHG emissions produced in the entire life cycle of the plants are considered including the construction and end-of-life phase [32].

Concerning the GHG emissions of the cradle-to-gate analysis carbon dioxide is the most significant GHG, see Table 2. Methane emissions are mainly caused by natural gas supply.

**Table 2:** Life cycle inventory (LCI) results of the NG-Case following the cradle-to-gate approach

Greenhouse gas	[kg/kg hot-rolled coil]
Carbon dioxide	1.3
Methane	1.3e-3

### Life Cycle Impact on Climate Change

**Fig. 4:**
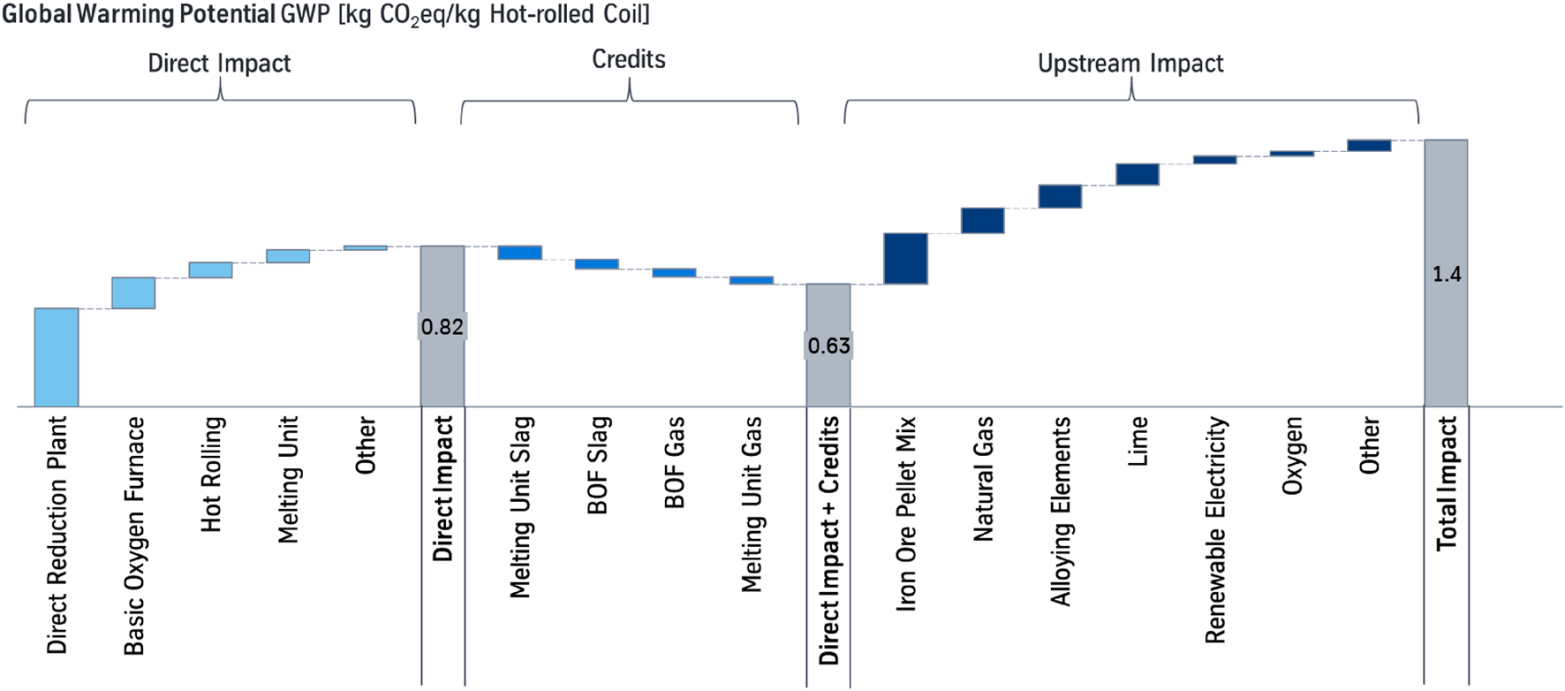
Global warming potential (GWP) of hot-rolled coil, produced over a natural gas-based direct reduction with an integrated electric melting unit (NG-Case). The respective system boundaries are referred to Fig. 3. The data are derived from internal communication of tkSE, year 2018–2020

Already in this scenario, the carbon footprint of hot-rolled coil reduces remarkably to 1.4 kg CO_2_eq/kg hot-rolled coil, see Fig. 4.

Since the reduction of the iron ores is shifted from coal to natural gas the direct impact of the integrated site is more than halved compared to the Base Case leading to a GWP of 0.82 kg CO_2_eq/kg HRC. While in the Base Case electricity created a surplus, this credit turns into a burden for electricity supply. The direct impact and the credits sum up to a GWP of 0.63 kg CO_2_eq/kg HRC.

This positive effect is narrowed by an increasing upstream impact. The DR plant is exclusively fed with iron ore pellets, which production accounts for the highest part of the upstream impacts. Yet, in sum the total GWP decreases significantly compared to the Base Case. Without consideration of a credit for the slag from the melting unit and from the BOF the GWP would be 1.5 kg CO_2_eq/kg HRC.

The improvement of the GWP compared to the Base Case is based on a shift from using coal for reducing and melting the iron ores towards using natural gas for reducing and renewable electricity for melting the iron ores. If no renewable electricity is used but a German or European grid mix the GWP increases up to 1.7 kg CO_2_eq/kg HRC, see Table 3.

**Table 3:** Carbon footprint of hot-rolled coil as a function of the electricity mix

Electricity mix Input	Carbon footprint of electricity mix [kg CO_2_eq/kWh]	Carbon footprint of hot-rolled coil [kg CO_2_eq/kg HRC]
German renewable Mix^a^	0.056	1.4
German grid mix^b^	0.54	1.7
European grid mix^c^	0.39	1.6

The electricity mix is used for the DR plant, melting unit, BOF, casting, and natural gas-based hot-rolling.

^a^German renewable electricity mix, year 2018 according to the Environment Agency [34]; GaBi database, 2021.1: “DE: Electricity mix (energy carriers, generic)”.

^b^GaBi database, 2021.1: “DE: Electricity grid mix”

cGaBi database, 2021.1: “EU-28: Electricity grid mix”

## Product Carbon Footprint for a Hydrogen-Based Direct Reduction Plant with an Integrated Electric Melting Unit

### Goal and Scope

DR plants allow a stepwise transition from natural gas towards hydrogen input. The potential of an only hydrogen operation is discussed in the following.

The system boundary of the H_2_-Case is in accordance to the NG-Case (Fig. 3). Instead of natural gas hydrogen is used for the DR plant. It is assumed that the hydrogen is from electrolysis driven by a renewable electricity mix. No credit is given for the co-product oxygen of the electrolysis process. The gas preheater of the DR plant is electrified as well as the slab heating, see Fig. 3. The carbon content of the DRI would be zero when using pure hydrogen as reducing gas in the DR plant. Since in the electric melting unit the DRI should be further reduced and carburized to hot metal, coal is added in the electric melting unit. It is assumed that the C-content of the hot metal is adjusted from typically 4.5% C to 2.0% C, since the carbon input would be minimized in case of an only hydrogen reduction. The DRI is charged hot into the electric melting unit.

Likewise in the NG-Case it is assumed that the off-gases from the electric melting unit and the BOF are used for thermal heat supply and thus credits for natural gas substitution are given. In the long-term these credits may not be justified anymore and credits for renewable hydrogen supply would be rather appropriate instead. The slag from the electric melting unit is assessed as a cement substitute. With decreasing environmental impacts of the cement industry in the long-term these credits will decrease, as well. Therefore also the GWP of hot-rolled coil without consideration of credits is communicated.

### Life Cycle Inventory

The data for the H_2_-Case are taken from internal communication of tkSE (2020). The iron and energy feedstock of the integrated steel site are listed in Table 4. The defined cut-off criteria are described in the NG-Case.

**Table 4:** Major inputs of the integrated steel site for the H_2_-Case

Input	[unit input/kg hot-rolled coil]
Iron ore pellets (kg)	1.5
Scrap (kg)	0.2
Coal (kg)	0.039
Electricity (MJ)	5.7
Hydrogen (MJ)^a^	6.9

^a^Related to LHV (120 MJ/kg)

The major energetic inputs are electricity and hydrogen. Imported electricity is modelled as a German renewable electricity mix from the year 2018 according to the Environment Agency [34]. The hydrogen is assumed to be produced from the same renewable electricity mix via water electrolysis [27]. Thus, in sum 17 MJ/kg hot-rolled coil of electric energy are required. According to a GaBi database for an electrolysis process,^4^ an electricity demand of 192 MJ/kg H_2_ is needed, which is equivalent to an efficiency of 62.5% [lower heating value (LHV) of hydrogen/energy unit of electricity].

Concerning the GHG emissions of the cradle-to-gate analysis carbon dioxide is the most significant GHG, see Table 5. The methane emissions are mainly caused by the renewable electricity. The renewable energy input contains electricity production from biogas. Thereby fugitive methane emissions are emerged.

**Table 5:** Life cycle inventory (LCI) results of the H_2_-Case following the cradle-to-gate approach

Greenhouse gas	[kg/kg hot-rolled coil]
Carbon dioxide	0.63
Methane	3.4e-3

### Life Cycle Impact on Climate Change

**Fig. 5:**
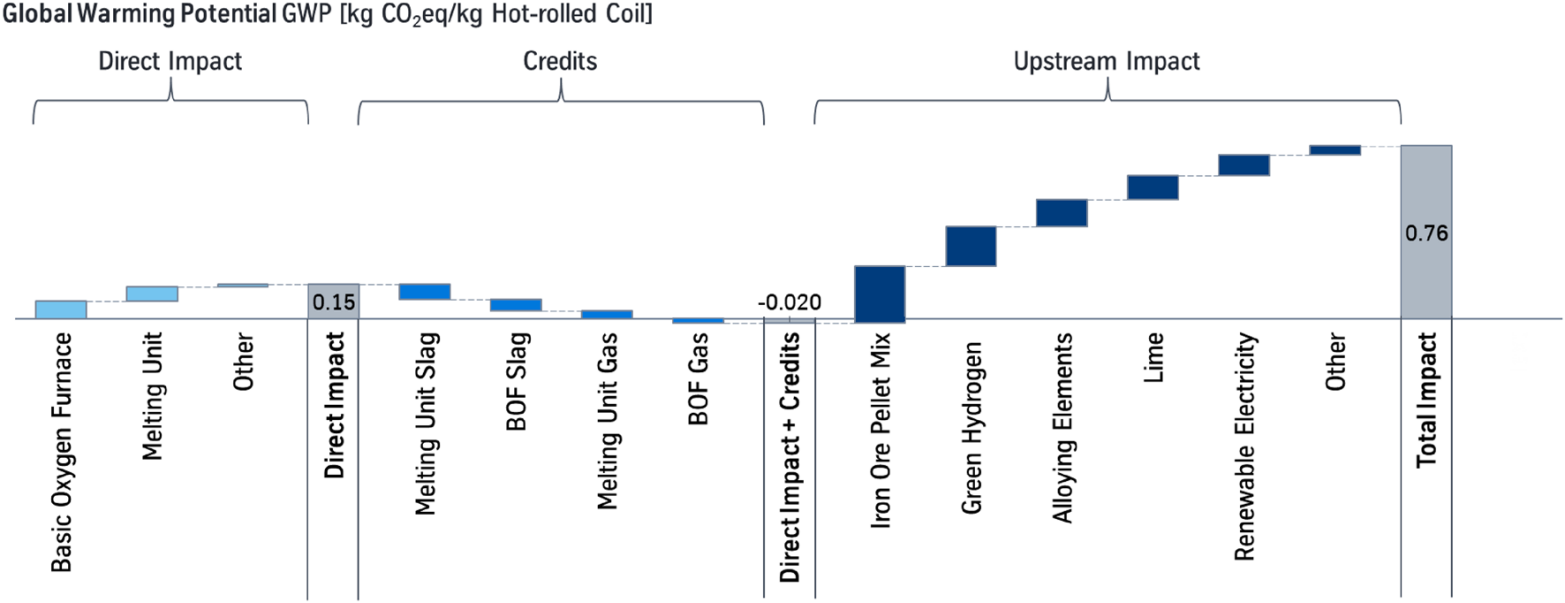
Global warming potential (GWP) of hot-rolled coil, produced over a hydrogen-based direct reduction with an integrated electric melting unit (H_2_-Case). The respective system boundaries are referred to Fig. 3. The data are derived from internal communication of tkSE, year 2018–2020

In the concluding H_2_-Case the carbon footprint is further reduced to 0.76 kg CO_2_eq/kg HRC, see Fig. 5.

The main impact on climate change is caused by the upstream processes, which add up to 0.78 kg CO_2_eq/kg HRC. The iron ore input in form of exclusively pellets causes the highest part of the upstream processes leading to a GWP of about 0.25 kg CO_2_eq/kg HRC. In addition, the alloying elements and burnt lime have a significant impact on the total GWP. Besides the material input, the imported renewable electricity mix as well as the indirectly required electricity for the hydrogen electrolysis lead to a GWP of about 0.26 kg CO_2_eq/kg HRC. The hydrogen has a specific footprint of 3.06 kg CO_2_eq/kg H_2_ [27]. These impacts are based on data referring to a time span between year 2018 and 2020. These results demonstrate that besides the processes of an integrated steel site, also the environmental impacts of upstream processes and renewable electricity supply will need to be reduced.

Emissions from incineration of the BOF gas and the melting unit gas generate a GWP of 0.15 kg CO_2_eq/kg HRC. Thus, there is still a direct impact of the integrated steel site, since it still depends on carbon. It maintains that the steel industry may rely on a biogenic carbon source in the future. The GWP without consideration of credits is 0.93 kg CO_2_eq/kg HRC.

The improvement of the GWP compared to the Base Case and the NG-Case results from the fact that the iron ores are reduced with hydrogen, which results from renewable electricity, and melted with renewable electricity. If no renewable electricity is assumed but a European or German grid mix, the carbon footprint of the HRC increases up to 2.3 or 3.0 kg CO_2_eq/kg HRC, see Table 6. This underlines the importance for the availability of renewable electricity.

**Table 6:** Carbon footprint of hot-rolled coil as a function of the electricity mix for the H_2_-Case

Electricity mix Input	Carbon footprint of electricity mix [kg CO_2_eq/kWh]	Carbon footprint of hot-rolled coil [kg CO_2_eq/kg HRC]
German renewable mix^a^	0.056	0.76
German grid mix^b^	0.54	3.0
European grid mix^c^	0.39	2.3

The electricity mix is used for the water electrolysis, DR plant, melting unit, BOF, casting, and electrified hot-rolling.

^a^German renewable electricity mix, year 2018 according to the Environment Agency [34]; GaBi database, 2021.1: “DE: Electricity mix (energy carriers, generic)”.

bGaBi database, 2021.1: “DE: Electricity grid mix”

cGaBi database, 2021.1: “EU-28: Electricity grid mix”

A comparison of Tables 3 and 6 results in a break-even point of 0.21 kg CO_2_eq/kWh electricity. Below this carbon footprint, the use of hydrogen from electrolysis is superior to the use of natural gas regarding to the impact on climate change.

### Energy Transformation of the Steel Industry

In the following, the energy transformation of a coal-based conventional integrated steel site towards a hydrogen- and electricity-based integrated site is summarized and discussed based on the results of the life cycle inventories. In all scenarios an equal input of scrap is assumed.

**Fig. 6:**
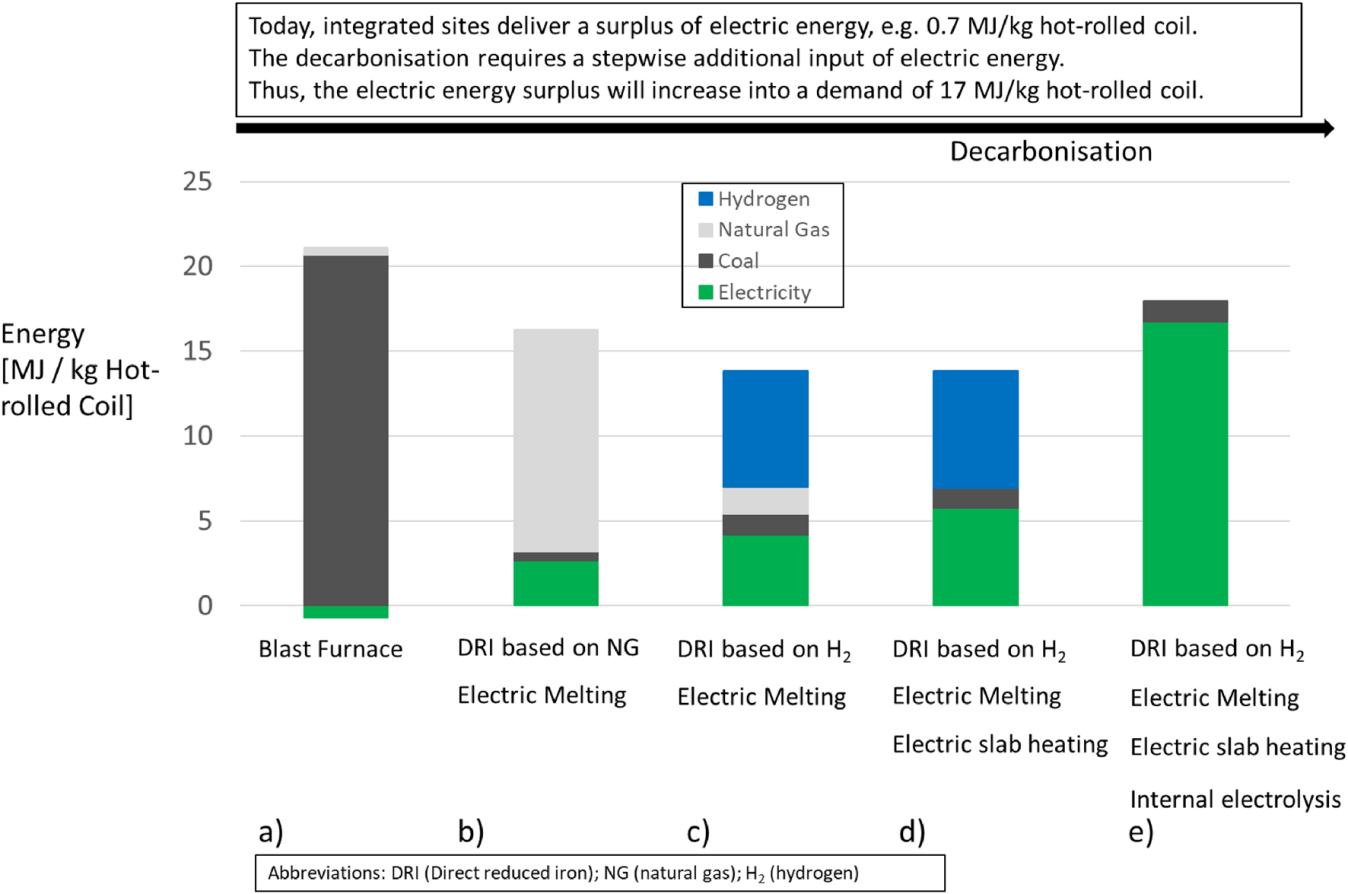
Future energy demand of an integrated steel mill

Figure 6 gives a detailed overview on the changing energy demands (LHV) of the integrated steel site in subsequent steps.

- *Base Case* The demand of energy and reducing agents of a conventional BF-BOF route is almost exclusively provided by coal (21 MJ/kg HRC). The energy demand of natural gas is 0.43 MJ/kg HRC. The pyrolysis of coal into coke and the reduction of iron ores by coal and coke leads to process gases, which provide heat and electricity for the integrated site. Surplus electricity is even exported as part of national grids. The direct energy demand for the processes sinter plant, coke oven, blast furnace, BOF, casting, hot-rolling, and energy output from the power plant is presented (Fig. 6, pillar a). The carbon footprint of this scenario is presented in Fig. 2.
- The iron ores are directly reduced by natural gas in a DR plant and subsequently melted in an electric melting unit. The DRI is charged hot into the melting unit. DR plants convert integrated sites from electricity producers to electricity consumers. Small amounts of coal (0.015 kg/kg HRC) are still added in the electric melting unit to further reduce the wustite of the DRI into iron and to carbonize the iron into hot metal. The reduction of the wustite improves the FE-yield of the process chain. The direct energy demand for the processes DR plant, electric melting unit, BOF, casting, and hot-rolling is presented. The carbon footprint of this scenario is presented in Fig. 4.
- The direct reduction is completely based on hydrogen. The preheating of the hydrogen is electrified. The DRI is also charged hot into the electric melting unit. As DRI from hydrogen reduction is carbon free, some extra carbon (0.039 kg coal/kg HRC) has to be introduced to promote beneficial metallurgical reactions. Natural gas is used for slab heating within the hot-rolling processes. The direct energy demand for the processes DR plant, electric melting unit, BOF, casting, and hot-rolling is presented.
- As a further step, the slab heating of the hot-rolling process is electrified. The direct energy demand for the processes DR plant, electric melting unit, BOF, casting, and hot-rolling is presented. The carbon footprint of this scenario is presented in Fig. 5.
- Finally the energy demand for hydrogen is translated into a need for electricity matching on-site electrolysis. A constant efficiency of 62.5% (LHV of hydrogen / electricity demand of electrolysis)^5^ is assumed. The direct energy demand for the processes electrolysis, DR plant, electric melting unit, BOF, casting, and hot-rolling is presented. The carbon footprint of this scenario is the same as in d), since the carbon footprint assessment is a cradle-to-gate analysis and thus the step from electricity to hydrogen production is included.

The decrease of the energy demand from the Base Case (a) to the NG-Case (b) has several reasons:

- A blast furnace consumes about 15 MJ energy in form of coal and coke to produce one kg of hot metal. Via a DR plant combined with electrically melting, only 13 MJ are required to produce one kg of hot metal. The process gas of the DR plant is recirculated within a close loop. The blast furnace gas is used for thermal heat and is electrified, whereby in both steps energy is dissipated.
- A coke plant is needed for the operation of a blast furnace. If natural gas is used for the direct reduction, no respective upstream process is needed.
- Sinter is used as an iron feedstock within a blast furnace. The sintering process consumes energy, mainly in form of coke. About 1.6 MJ energy per kg of sinter is required. A direct reduction plant is fed with iron ore pellets or lump ore. The pelletizing process is outside the system boundaries for the energy-related consideration of Fig. 6. Anyway, the process of pelletizing is less energy-intensive than the sintering process and using directly lump ore instead of pellets is a possibility, as well.

For decarbonizing the steel industry the energy surplus of a conventional integrated site will increase into a demand of 17 MJ per kg of HRC, which is equal to 4.7 kWh/kg HRC. This electric energy has to be delivered by renewable energies.

Within the European Union (28) an absolute amount of 159 million tonnes of steel is produced in year 2019^6^ [35]. The share of the BF-BOF route is 59% leading to about 94 million tonnes of steel produced via the BF-BOF route in EU (28) in year 2019 [35]. The transformation from the BF-BOF route towards climate-neutral steel production will most likely be performed via hydrogen-based direct reduction combined with electrically melting. This production route is from technological readiness and scalability the leading technology alternative to the primary BF-BOF route [1]. Combining nowadays European primary steel production [35] and the results from Fig. 6, a shift from present European coal-based steel production towards an electrically based steel production would lead to an electricity demand of about 440 TWh per year for the European Union (28), an immense future challenge.

## Conclusions

Expected future demand of steel suggests that integrated steel mills will continue to produce steel far beyond the year 2050 from iron ore. As a pre-condition integrated sites have to become significantly CO_2_eq-reduced: Coal-based reduction of ore needs to be replaced by carbon-reduced techniques. Modern DRI plants are technical ready and capable to support such a transition away from coal towards natural gas and subsequently hydrogen. Although a pure hydrogen-based shaft furnace direct reduction process in a large scale has not been realized yet the concept is technically feasible and has been proven for hydrogen-rich operation modes.

Low GHG-intensive steel production requires electrically melting of the direct reduced iron. Any use in blast furnaces or as scrap substitute in BOFs can only be a transition step. After electrically melting a pre-melt of DRI/HBI can either still pass the BOF or already be used as raw steel. The decision depends on the product portfolio—many of today’s chemical steel compositions require subsequent BOF treatment.

Whereas there are plenty of LCA and PCF studies about conventional steel production via the BF route there is a lack of studies for future steel production via a DR plant and electrically melting. This study fills this gap by providing a holistic carbon footprint assessment according to ISO 14067 for steel, produced via direct reduction, electrically melting, and subsequent refining in a BOF. The carbon footprint assessments for all considered scenarios are based on the same methodologies and databases; so these have an impact on the absolute values but their sensitivity on the deltas between these scenarios is much weakened.

The actual value of traditional coal-based steel production causes a global warming potential of 2.1 kg CO_2_eq/kg HRC. As a transition scenario, natural gas-based direct reduction can reduce remarkably the global warming potential to 1.4 kg CO_2_eq/kg hot-rolled coil. With hydrogen-based direct reduction the carbon footprint can further be reduced to 0.76 kg CO_2_eq/kg hot-rolled coil.

The most significant driver is the carbon footprint of the electricity mix, which is used for water electrolysis and directly for the processes of the integrated steel site. If no renewable electricity is available and e.g., the current European electricity mix has to be used the carbon footprint of steel can even increase compared to the BF-BOF route.

Until 2050, the energy surplus of an integrated site will increase into a demand of 17 MJ per kg of hot-rolled coil, which is equal to 4.7 kWh/kg HRC. In order to reach a fossil-free steel production, this electric energy has to be delivered by renewable energy, an immense future challenge.

Limitations of the study are that the data for the future scenarios are based on metallurgical models. If primary data are available this paper can be extended to a life cycle assessment (LCA) considering more environmental impact categories than climate change. Since the used data of this paper are confidential company data, no complete inventory data set is presented. Thus, the reproducibility is limited. Within a life cycle sustainability assessment (LCSA) also economic and social pillars could be analyzed. Concerning the assessment of co-products the methodology of system expansion is used. Credits are given in dependency of the environmental impacts of the substituted primarily produced products. For future scenarios these values have a degree of uncertainty. That’s why, the results are also presented without consideration of credits. Emissions from collecting, sorting and processing of scrap are not considered in this paper. In addition, emissions from the construction phase of facilities, machines, and infrastructure of the integrated steel site are not included.

## Supplementary Information

Below is the link to the electronic supplementary material.

Supplementary file1 (PDF 194 kb)

## Abbreviations

BF: Blast furnace
BOF: Basic oxygen furnace
CO: Carbon monoxide
CO_2_: Carbon dioxide
CO_2_eq: Carbon dioxide equivalent
DR: Direct reduction
DRI: Direct reduced iron
EAF: Electric arc furnace
GHG: Greenhouse gas
GWP: Global warming potential
H_2_: Hydrogen
HBI: Hot briquetted Iron
HRC: Hot-rolled coil
IPCC: Intergovernmental panel on climate change
LCA: Life cycle assessment
LCI: Life cycle inventory
LHV: Lower heating value
NG: Natural gas
PCF: Product carbon footprint
tkSE: Thyssenkrupp Steel Europe AG

## Footnotes

1 The Base Case of the previous study [27] is also used as Base Case for this study. The previous study was done by the same authors of this study so the same methodological approach was followed.

2 GaBi database, 2021.1 (DE: BF Steel billet/slab/bloom).

3 incl. the Base Case (Fig. 2), which was done by the same authors of this paper [27].

4 GaBi database, 2021.1: “GLO: Hydrogen (electrolysis, decentral – for partly aggregation, open input electricity)”.

5 GaBi database, 2021.1: “GLO: Hydrogen (electrolysis, decentral – for partly aggregation, open input electricity)”.

6 Refer to year 2019 to diminish Covid-19 effects.
