# Supplementary material for: Carbon Footprint and Energy Transformation Analysis of Steel Produced via a Direct Reduction Plant with an Integrated Electric Melting Unit
Source: J Sustain Metall. 2022 Aug 31;8(4):1532–45. doi: 10.1007/s40831-022-00585-x (PMC9430015; doi:10.1007/s40831-022-00585-x)
Supplement: Supplementary file 1 — Supplementary file1 (PDF 194 kb) [file 40831_2022_585_MOESM1_ESM.pdf]

Supplementary Material for *Journal of Sustainable Metallurgy*

Carbon Footprint and Energy Transformation Analysis of Steel Produced via a Direct Reduction Plant with an Integrated Electric Melting Unit

Julian Suer, Frank Ahrenhold, Marzia Traverso

Cut-off criteria and assumptions

The Worldsteel Association defined the following cut-off criteria for steel production [1]:

- All energetic input flows must be included.
- Each excluded material flow must not exceed 1% of mass, energy or environmental relevance for each process.
- The sum of the excluded material flows in the system must not exceed 5% of mass, energy or environmental relevance.

The raw materials iron ore, pellet, coke, coal, scrap, limestone, lime, and dolomite represent more than 95% of the total mass input (except water) [1]. These raw materials are all considered in this paper. Additionally, the emission-intensive alloying elements and also the less emission-intensive inputs like gravel, bauxite, graphite amongst others are included. All energy related inputs are considered. Thus, the cut-off criteria are conform to those defined by the Worldsteel Association [1]. The used GaBi databases [2] for secondary data are listed in table 1, table 2, table 3, table 4, and table 5.

Internal transport is neglected in this study. According to the Worldsteel Association the emissions from internal transport are 0.00024 kg CO_2/kg crude steel [3]. Thus these emissions can be cut-off according to the defined cut-off criteria. The construction phase of facilities, machines, and infrastructure of the integrated steel site are not considered. The emissions must be spread along their life span and are thus low compared to the process emissions of the energy-intensive steel production. However, the construction phase of the renewable electricity processes, which are especially for the H_2-Case of the study relevant, are included by the GaBi databases [2].

GaBi databases for secondary data

The used GaBi databases for secondary data are listed in the following. In table 1 the databases for the raw materials are listed; in table 2 the databases for the energy-related inputs; in table 3 the databases for the evaluation of the co-products; in table 4 the databases for the hydrogen production from electrolysis driven by a renewable electricity mix; in table 5 the databases for the input of alloying elements:

Table 1: *GaBi databases for raw material inputs.*

Material Flows	GaBi Database (2021.1)
**Steel Production - Material Flows**	
Argon	DE: Argon (gaseous)
Bauxite	EU-28: Bauxite
Calcium hydroxide	DE: Calcium hydroxide
Cement	EU-28: Cement (CEM I 42.5)
Compressed air	GLO: Compressed air 7 bar
Dolomite	DE: Dolomite (ground)
Graphite	DE: Synthetic Graphite (via Petrol coke)
Iron Ore	DE: Iron ore-mix

Iron Ore Pellets	DE: Pellet-feed-mix (import mix)
Landfill	DE: Landfill for inert matter (Steel)
Limestone	DE: Limestone ($CaCO_3$; washed)
Lubricant	DE: Lubricants at refinery
Magnesium	CN: Magnesium
Nitrogen	DE: Nitrogen (gaseous)
Oxygen	DE: Oxygen (gaseous)
Process Water	EU-28: Process water
Quicklime	DE: Lime (CaO; finelime)
Sodium Chloride	DE: Sodium chloride (rock salt)
Silica Sand	DE: Silica sand (Excavation and processing)
Water (deionised)	DE: Water (desalinated; deionised)
Water (from groundwater)	EU-28: Tap water from groundwater

Table 2: *GaBi databases for energy-related inputs.*

Material/Energy Flows	GaBi Database (2021.1)
**Steel Production - Energy Flows**	
District Heating	EU-28: District heating mix
Electricity	DE: Electricity grid mix
Hard coal mix	DE: Hard coal mix
Hydrogen	see Table 4
Natural Gas	DE: Natural gas mix
Steam	DE: Process Steam from natural gas, 95%

Table 3: *GaBi databases for co-product evaluation.*

Material/Energy Flows	GaBi Database (2021.1)
**Steel Production – Co-products**	
Benzene	DE: Benzene mix
Electricity	DE: Electricity mix
District heating	EU-28: District heating mix
Slag from basic oxygen furnace	DE: Lime (CaO; finelime)<br>EU-28: Gravel 2/32
Slag from blast furnace and from melting unit	Cement Mixer (Worldsteel)<br>EU-28: Gravel 2/32<br>DE: Landfill for inert matter
Sulphur	DE: Sulphur (elemental) at refinery
Tar	EU-28: Bitumen at refinery

Table 4: *GaBi databases for hydrogen production.*

Material/Energy Flows	GaBi Database (2021.1)
**Hydrogen Production**	
Electrolysis Process	GLO: Hydrogen (electrolysis, decentral)
**Electricity for Electrolysis from**	
Biomass	DE: Electricity from biomass (solid)
Biogas	DE: Electricity from biogas
Hydro	DE: Electricity from hydro power
Geothermal	DE: Electricity from geothermal
Photovoltaic	DE: Electricity from photovoltaic
Wind	DE: Electricity from wind power

Table 5: *GaBi databases for alloying elements.*

Material Flows	GaBi Database (2021.1)
**Steel Production - Alloying Elements**	
Aluminium	DE: Aluminium ingot mix
Calcium silicate	EU-28 Calcium silicate
Copper	DE: Copper mix (99.999% from electrolysis)
Ferro Chrome Mix	DE: Ferro chrome mix (60%)
Ferro Manganese Mix	ZA: Ferro-manganese mix (74-82%)
Ferro Molybdenum	GLO: Ferro molybdenum(70-90%)
Ferro Silicon	GLO: Ferro silicon mix (90% Si)
Ferro Vanadium	ZA: Ferro-Vanadium
Nickel	GLO: Nickel mix (99.9%)
Tin	GLO: Tin
Titanium	GLO: Titanium
Titanium dioxide (rutile)	EU-28: Titanium dioxide pigment

References

[1] World Steel Association (2017): Life Cycle Inventory Methodology Report.
[2] ©Sphera Solutions GmbH (2021): Gabi Database 2021.1
[3] World Steel Association (2019): Life Cycle Inventory Study. 2019 data release.
